# Newborn Screening: Current Status in Alberta, Canada

**DOI:** 10.3390/ijns5040037

**Published:** 2019-10-01

**Authors:** Andy De Souza, Vanessa Wolan, Angie Battochio, Susan Christian, Stacey Hume, Grace Johner, Margaret Lilley, Ross Ridsdale, Kareena Schnabl, Chi Tran, Jolene Yuen-Jung, Iveta Sosova

**Affiliations:** 1 Newborn Metabolic Screening and Biochemical Genetics Laboratory, University of Alberta Hospital, Alberta Public Laboratories, Edmonton, AB T6G 2B7, Canada; Andy.DeSouza@albertapubliclabs.ca (A.D.S.); Vanessa.Wolan@albertapubliclabs.ca (V.W.); Angie.Battochio@albertapubliclabs.ca (A.B.); Ross.Ridsdale@albertapubliclabs.ca (R.R.); Kareena.Schnabl@albertapubliclabs.ca (K.S.); Chi.Tran@albertapubliclabs.ca (C.T.); Jolene.Yuen-Jung@albertapubliclabs.ca (J.Y.-J.); 2 Department of Laboratory Medicine and Pathology, University of Alberta, Edmonton, AB T6G 2B7, Canada; 3 Molecular Diagnostics Laboratory, University of Alberta Hospital, Alberta Public Laboratories, Edmonton, AB T6G 2B7 Canada; Susan.Christian@albertapubliclabs.ca (S.C.); Stacey.Hume@albertapubliclabs.ca (S.H.); Margaret.Lilley@albertapubliclabs.ca (M.L.); 4 Department of Medical Genetics, University of Alberta, Edmonton, AB T6G 2H7, Canada; 5 Screening Programs, Alberta Health Services, Calgary, AB T2S 3C3, Canada; Grace.Johner@albertahealthservices.ca

**Keywords:** newborn screening, Alberta, second-tier

## Abstract

Newborn screening (NBS) in Alberta is delivered by a number of government and health service entities who work together to provide newborn screening to infants born in Alberta, the Northwest Territories, and the Kitikmeot region of the Nunavut territory. The Alberta panel screens for 21 disorders (16 metabolic, two endocrine, cystic fibrosis, severe combined immunodeficiency, and sickle cell disease). NBS is a standard of care, but is not mandatory. NBS performance is monitored by the Alberta Newborn Metabolic Screening (NMS) Program and NMS Laboratory, who strive for continuous quality improvement. Performance analysis found that over 99% of registered infants in Alberta received a newborn screen and over 98% of these infants received a screen result within 10 days of age.

## 1. Introduction

Newborn screening (NBS) has dramatically improved the morbidity and mortality associated with the screened disorders. NBS is the first and largest example of systematic, population-wide genetic testing [1]. The history of newborn screening began in the 1960s when Robert Guthrie introduced the Guthrie card and a bacterial inhibition assay to screen for phenylketonuria (PKU) in dried blood spots [2]. During the 1970s and the 1980s, the additions of new screening conditions were limited by the available technology, which restricted screening to one-analysis, one-metabolite, one-disease assays. In the 1990s, the introduction of tandem mass spectrometry (MS/MS) into NBS laboratories revolutionized the NBS field, as this technology had the ability to screen for several different disorders by simultaneous amino acid and acylcarnitine analyses in a single blood spot [3,4,5,6,7,8,9]. As a result, NBS panels began rapidly expanding to include additional inborn errors of amino acid, organic acid, and fatty acid metabolism. Further expansion of screening panels in the 2000s introduced molecular genetic methods and point-of-care testing into newborn screening [10,11,12].

NBS in Canada originated in the 1960s, shortly after Guthrie introduced the concept of PKU screening. Canada is the second largest geographical country in the world and has a population of 35.19 million [13]. The country is divided into 10 provinces and three territories. Health care is publicly funded and falls under the provincial or territorial jurisdiction; therefore, newborn screening is managed provincially or territorially. Unfortunately, neither the screening panels nor the approaches to calculating the number of conditions included in the screening panels are harmonized within the country. Consequently, the disorders screened depend on where an infant is born and can vary from 12 to 40 disorders [14,15,16,17,18,19,20,21,22]. 

**Figure 1:**
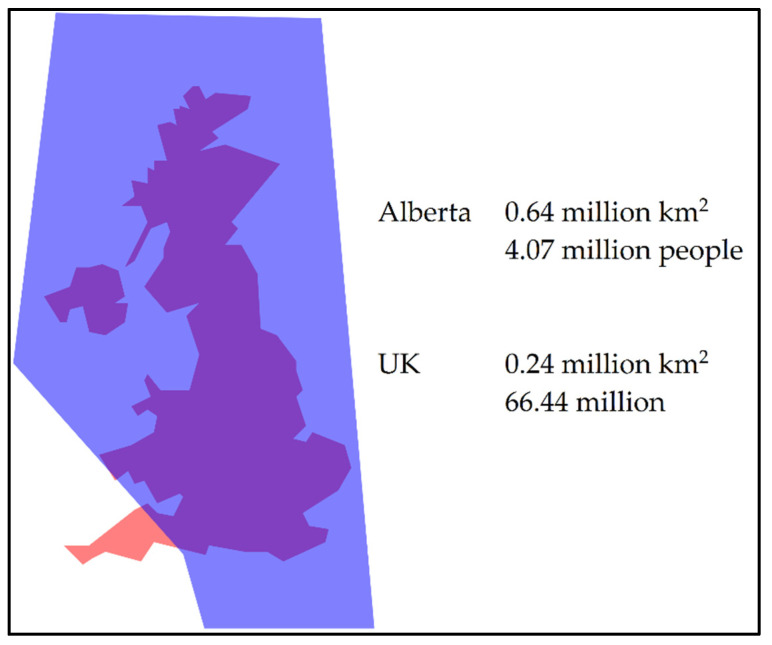
Geographical comparison of the province of Alberta, Canada (blue) to the United Kingdom of Great Britain and Northern Ireland (orange) [13,23,24].

The province of Alberta is approximately 2.5 times greater in area than the United Kingdom of Great Britain and Northern Ireland (UK) (Figure 1); however, Alberta’s population (4.07 million) is approximately 16 times less than that of the UK [13,23]. Access to health care in Alberta has geographical challenges, with regions that are northern and rural. 

## 2. Alberta Newborn Metabolic Screening 

The Alberta Newborn Metabolic Screening (NMS) Program is delivered by Alberta Health Services (AHS), including Alberta Public Laboratories (APL), in partnership with Alberta Health, Indigenous Services Canada’s First Nations Inuit Health Branch, physicians and midwives, as well as parents or guardians. Although referred to as Newborn Metabolic Screening in Alberta, the disorders screened are not exclusively metabolic disorders. 

The Alberta NMS Laboratory is located at the University of Alberta Hospital (UAH) in the province’s capital, Edmonton, and provides screening to infants born in Alberta, the Northwest Territories, and the Kitikmeot region of the Nunavut territory. The AHS Provincial NMS Program coordinates the operational aspects of newborn screening in Alberta and utilizes a quality management framework to support the screening process. To assist with this process, Alberta launched a secure, web-based application in 2000, called NMS Application, for electronic tracking of the screening status. 

Newborn screening in Alberta was implemented in 1967 with the introduction of screening for PKU, which was supplemented in 1977 and in 1990 with congenital hypothyroidism (CH) and biotinidase deficiency (BIOT) screening, respectively. In 2007, 14 disorders were added to the NMS panel: carnitine uptake defect (CUD), citrullinemia (CIT), congenital adrenal hyperplasia (CAH), cystic fibrosis (CF), glutaric acidemia type 1 (GA1), 3-hydroxy-3-methylglutaryl-CoA lyase (HMG) deficiency, isovaleric acidemia (IVA), long chain hydroxyacyl-CoA dehydrogenase (LCHAD) deficiency, maple syrup urine disease (MSUD), medium chain acyl-CoA dehydrogenase (MCAD) deficiency, methylmalonic acidemia (MMA), propionic acidemia (PA), trifunctional protein (TFP) deficiency, and very long chain acyl-CoA dehydrogenase (VLCAD) deficiency. Alberta was the first jurisdiction in Canada to introduce CF to its screening panel [25]. Further additions to the panel occurred recently in 2019 with the addition of four disorders: classic galactosemia (GALT), tyrosinemia type 1 (TYR1), severe combined immunodeficiency (SCID), and sickle cell disease (SCD). Alberta is the third jurisdiction in Canada to offer newborn screening for SCID, following Ontario in 2013 and the Maritimes (New Brunswick, Nova Scotia, and Prince Edward Island) in 2016 [19,21]. As of 2019, Alberta screens for 21 treatable disorders: 16 metabolic disorders, two endocrine disorders, CF, SCID, and SCD. The disorders are screened by the NMS Laboratory, with the exception of SCID and second-tier testing for CF, which are performed by the Molecular Diagnostics Laboratory at the UAH. CF was the first assay on Alberta’s NMS panel to utilize a two-tier screen. The first tier is immunoreactive trypsinogen and the second tier is a molecular test for common pathogenic variants in the cystic fibrosis transmembrane conductance regulator (CFTR) gene. A summary of the screened conditions and methodologies is shown in Table 1. 

## 3. Screening Process 

In Alberta, newborn screening is voluntary; parents or guardians can exercise refusal of screening. Consent for newborn screening is verbal; however, refusal of screening must be documented by the health professional in accordance with the NMS Program policy. 

**Figure 2:**
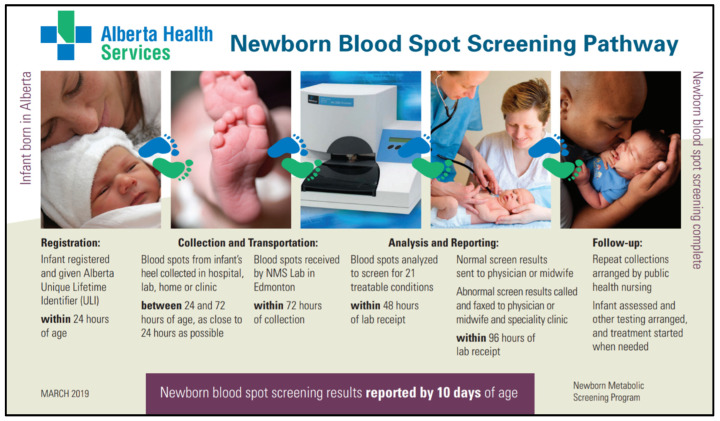
Example of educational material describing the newborn blood spot screening pathway.

The newborn screening process begins with infant registration and concludes with a negative screen result or referral to diagnostic and treatment services when a positive screen is identified (Figure 2). All infants born in Alberta are registered immediately after birth in the Person Directory (PD) application, which provides a unique lifetime identifier (ULI). The infant’s ULI is provided on the NMS requisition and is used to track the sample through the NMS Application. Both the PD and the NMS Application are maintained by Alberta Health. The NMS Application is integrated with the PD and the NMS Laboratory Information System (LIS) in order to track infants that are born in Alberta and their subsequent newborn screens. Furthermore, it is used to coordinate follow-up, both screening and clinical.

**Figure 3:**
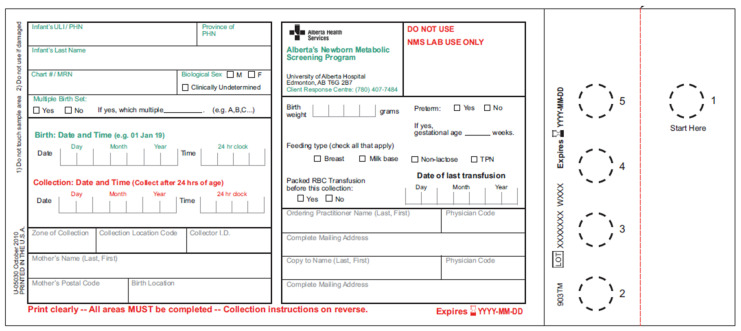
Newborn blood spot screening requisition with a detachable section, allowing for simultaneous testing in two laboratories.

Collection of the blood specimens is recommended at 24 to 72 h of age, ideally closer to 24 h. The upper age limit for screening is 24 months, allowing the inclusion of infants from international adoptions and infants from new immigrant and refugee families. In addition to demographic data, the NMS requisition includes a field for information on gestational age, birth weight, feeding type, and transfusion status (Figure 3). If a newborn requires a blood transfusion, the recommendation is to collect the specimen before the transfusion, even if the infant is less than 24 h old. Blood from the infant’s heel is collected on filter paper attached to the NMS requisition and the samples are air-dried for a minimum of three hours before transport. Each NMS requisition has a fold-over cover that protects the blood spots from contamination. One of the blood spots is located on a perforated portion of the card and this portion has a barcode identical to the requisition, allowing it to be separated from the original card and transported to the Molecular Diagnostics Laboratory for SCID testing. NMS samples are collected in a variety of health care centers such as postpartum hospital units, neonatal intensive care units, special care nurseries, outpatient laboratory services, and at home by midwives or public health nursing. Often, samples require recollection due to illegible demographic information, quality of sample collection, or collection before 24 h of age. The NMS Application will generate alerts for these cases, which allows for the subsequent tracking and recollection of these samples. In the 2018–2019 reporting year, 1038 inadequate sample alerts (1.88%) were generated for 55,316 samples received, the latter including out-of-province samples and follow-up samples (Table 2). Nine hundred sixty-four (92.87%) of the inadequate samples required a repeat collection, of which 809 (89.92%) repeat collections occurred within 96 h of notification of the inadequate sample. The NMS Program provides feedback to collection sites in the form of quarterly sample quality reports to reduce the number of inadequate sample alerts. 

Samples are transported from across the province at ambient temperature in a sealed newborn screening envelope to the NMS Laboratory in Edmonton. The screening envelope is bright pink in colour for easy identification and has a space to indicate the number of specimens in the envelope, and that number is reconciled by the NMS Laboratory. A laboratory courier system and contracted courier services are used for transportation; standard mail is also occasionally used. Transport to the NMS Laboratory is to occur within 72 h of sample collection. Individual samples are not tracked during transport, but if a sample is not received by the NMS Laboratory within ten days of an infant’s birth, an alert is generated in the NMS Application.

Upon receipt in the NMS Laboratory, the specimens are barcoded and the demographic information, feeding type, and transfusion status from the requisition are entered into the LIS. The LIS communicates the demographic information to the NMS Application, where the birth registration is linked to the sample in order to track screening. The NMS Program monitors alerts and coordinates the collection of any missing samples. If a sample is received by the laboratory and a ULI has not been assigned to the infant, an alert is generated in the NMS Application and the NMS Program follows up with Alberta Health to ensure that the infant is registered. Samples are to be analyzed within 48 h and both normal and abnormal results reported within 96 h of receipt (including weekends and holidays) in the laboratory. For those samples requiring second-tier molecular genetic testing, results are to be reported within 21 days of receipt in the laboratory.

Samples with abnormal initial results are re-tested the next day for confirmation, with the exception of CF and GALT. There is no confirmation testing for CF. The confirmation and second-tier test for GALT is performed on the same day. The screening algorithms have two types of abnormal results: borderline or critical; however, not all disorders have borderline results. Borderline results on the initial specimen for BIOT, CUD, organic acidemias, and endocrine disorders are followed up with a second NMS collection. The NMS Application generates an alert for samples requiring recollection due to borderline results. If the second collection confirms the initial borderline result, the screen is called out as critical. Critical results require immediate follow-up by the responsible health care provider and the respective specialty clinic. Infants on total parenteral nutrition (TPN) may require a recollection 24 h post-discontinuation of TPN. All low birth weight (<2000 g) infants are recommended to have blood spots recollected at 21–28 days, even if the initial screen results are normal. Additionally, some premature infants with inadequate T cell development will be flagged for SCID re-testing 21–28 days after birth.

After completion of the screening, the NMS Laboratory LIS sends screening results to the NMS Application. If a result is not reported within 25 days of sample receipt by the NMS Laboratory, an alert is generated by the NMS Application for the laboratory to follow up on the testing status. Clinical follow-up for critical results is tracked in the NMS Application through alerts. The NMS samples are retained for 15 years, unless otherwise directed by the parent or guardian. Any additional non-newborn screening-related testing requires parent or guardian consent for sample use.

## 4. Alberta NMS 2019 Panel Expansion

The newborn screening panel was expanded to include GALT, SCD, and TYR1 on April 1, 2019, and SCID on May 31, 2019. 

GALT utilizes a two-tier screen, where the first tier measures GALT enzyme activity and the second tier measures total galactose concentration. The first tier is based on the Beutler spot assay and uses four enzymatic steps, including an initial GALT enzyme step [26]. A deficiency in any of these enzymes can result in an apparent decrease in GALT enzyme activity [27]. An advantage of the second tier, measuring total galactose, is that the incidental finding of galactose-6-phosphate dehydrogenase (G6PD) deficiency is not called out as a positive screen for classic galactosemia. 

Screening for SCD includes screening for sickle cell disease (HbSS), hemoglobin SC disease (HbSC), and hemoglobin S/beta thalassemia (HbSβ^−^ or HbSβ+). Sickle cell carriers (HbAS) are reported out as a positive sickle cell trait. All samples collected post-transfusion are sent for molecular genetic analysis.

The SCID screening generally follows the Wisconsin procedure [28]. An initial screen analyzing only the TREC copy number is performed and those that fall below 70 copies per microliter are duplicate screened using a multiplex assay which includes TREC and the RNaseP control gene. Those infants with adequate DNA and a TREC value below 40 copies per microliter are flagged for clinical follow-up.

TYR1 is screened by measuring succinylacetone using MS/MS.

## 5. Key Performance Measures and Outcomes from Alberta Newborn Screening

The NMS Program monitors and reports on the performance of newborn screening in Alberta using data generated from the NMS Application and the NMS Laboratory. Table 3 includes some of the performance measures reported by the NMS Program for the last three fiscal years. Over 99% of registered infants in Alberta received a newborn screen. The most common reason for not receiving a screen on a registered infant is neonatal death; other reasons include refusal by the parent or guardian or physician, inability to locate the infant, and relocation of the infant out-of-province. Over 98% of registered infants have results reported within 10 days of age. Approximately 29% of infants with abnormal screens receive an abnormal diagnostic outcome. In the 2018–2019 reporting year, 55 of 188 abnormal screens with follow-up completed were confirmed to have the associated disorder (Table 4). Diagnostic outcomes are communicated to the families by the respective specialty clinic, who also follow up with the patients, as required. The NMS Program is working towards implementation of tracking long-term outcomes of newborn screening.

## 6. Conclusions

The framework, design, and execution of the NMS Program is vital in ensuring that all infants born in Alberta are offered screening. Since the inception of newborn screening in Alberta in 1967 with screening for PKU, there have been several expansions, as recent as 2019, resulting in the current panel of 21 disorders. Due to disparity in newborn screening in Canada, infants’ health outcomes can be influenced by their province or territory of birth. The need for a national newborn screening strategy has been recognized. Despite meetings among the provinces and territories to create a national consensus, newborn screening panels remain non-uniform across Canada [29].

## Figures and Tables

**Table 1:** Alberta’s newborn screening panel.

Classification	Disorders	Screening Initiated	Assay and Instrumentation
**Metabolic Disorders—Organic Acid**	GA1	2007	PerkinElmer NeoBase™ Non-derivatized MSMS kit PerkinElmer NeoBase™ Succinylacetone Assay Solution Waters^™^ ACQUITY UPLC™ with XEVO^™^ TQD
	HMG deficiency	2007	
	IVA	2007	
	MMA	2007	
	PA	2007	
**Metabolic Disorders—Fatty Acid Oxidation**	CUD	2007	
	LCHAD deficiency	2007	
	MCAD deficiency	2007	
	TFP deficiency	2007	
	VLCAD deficiency	2007	
**Metabolic Disorders—Amino Acid**	CIT	2007	
	MSUD	2007	
	PKU	1967	
	TYR1	2019	
**Metabolic Disorders—Other**	BIOT	1990	PerkinElmer Neonatal Biotinidase kit PerkinElmer VICTOR2 ™D Instrument
	GALT	2019	PerkinElmer GSP^®^ Neonatal GALT kit PerkinElmer GSP^®^ Neonatal Total Galactose kit PerkinElmer GSP^®^ Instrument
**Endocrine Disorders**	CAH	2007	PerkinElmer AutoDELFIA^®^ Neonatal 17α-OH-progesterone kit PerkinElmer AutoDELFIA^®^ Instrument
	CH	1977	PerkinElmer AutoDELFIA^®^ Neonatal hTSH kit PerkinElmer AutoDELFIA^®^ Instrument
**Other Disorders**	CF	2007	PerkinElmer AutoDELFIA^®^ Neonatal IRT kit PerkinElmer AutoDELFIA^®^ Instrument Luminex^®^ xTAG^®^ CF39v2 Kit Luminex^®^ 200™
	SCID	2019	Laboratory developed test Applied Biosystems™ QuantStudio™ 12K Flex Real-Time PCR System
	SCD	2019	Bio-Rad VARIANT™ Sickle Cell Program kit Bio-Rad VARIANT™ nbs Newborn Screening System

**Table 2:** Summary of Newborn Metabolic Screening (NMS) Application inadequate sample alerts for the 2018–2019 reporting year. Reporting year is April 1 to March 31. Samples received, including out-of-province samples and follow-up samples, were 55,316.

Inadequate Sample Alert	Number	
Less than 24 h	172	(16.57%)
Blood clotted and layered	161	(15.51%)
Date or time of birth illegible or not indicated	2	(0.19%)
Date or time of collection illegible or not indicated	97	(9.34%)
Pre-expansion requisition	12	(1.16%)
Query patient identification	32	(3.08%)
Requisition expired	15	(1.45%)
Sample contaminated	186	(17.92%)
Sample insufficient	206	(19.85%)
Sample stability exceeded	1	(0.10%)
Specimen abraded	154	(14.84%)
Total	1038	

**Table 3:** Summary of NMS Program key performance measures. Reporting year is from April 1 to March 31.

Performance	2018–2019	2017–2018	2016–2017
Registered infants who received an initial blood spot screen ^1^	99.41% (52,005/52,313)	99.40% (52,898/53,215)	99.46% (54,891/55,190)
Registered infants who did not receive an initial blood spot screen	0.59% (308/52,313) 57.47% of infants not screened were due to neonatal death (177/308)	0.60% (317/53,215) 59.62% of infants not screened were due to neonatal death (189/317)	0.54% (299/55,190) 57.19% of infants not screened were due to neonatal death (171/299)
Registered screened infants who had a screen result reported within 10 days of age	98.74% (51,351/52,005)	99.15% (52,449/52,898)	99.22% (52,465/54,891)
Screened infants who received normal screen results ^2^	99.50% (51,837/52,099)	99.42% (52,649/52,958)	99.36% (54,608/54,960)
Screened infants who received abnormal screen results requiring clinical follow-up	0.36% (188/52,099) (162 critical results and 26 double borderline results)	0.44% (231/52,958) (200 critical results and 31 double borderline results)	0.49% (271/54,960) (250 critical results and 21 double borderline results)
Screened infants who received unknown screen results ^3^	0.14% (74/52,099)	0.15% (78/52,958)	0.15% (81/54,960)
Infants with abnormal screen result who received abnormal diagnostic outcomes	29.26% (55/188)	32.47% (75/231)	27.31% (74/271)
Infants with abnormal screen result who received likely to be normal diagnostic outcomes	55.85% (105/188)	61.47% (142/231)	68.63% (186/271)
Infants with abnormal screen result who received unclear diagnostic outcomes ^4^	1.60% ^5^ (3/188)	0.87% (2/231)	1.85% (5/271)
Infants with abnormal screen result who received unknown diagnostic outcomes ^6^	6.91% (13/188)	5.19% (12/231)	2.21% (6/271)
Infants with abnormal screen result with pending diagnostic outcomes	6.38% (12/188)	0.00% (0/231)	0.00% (0/271)

^1^ Infants born and registered in Alberta. ^2^ Includes infants born out-of-province and registered in Alberta. ^3^ An infant reported with inadequate, borderline, or TPN results has no confirmed normal or abnormal results on record. ^4^ Diagnostic test neither confirmed nor excluded the screened condition. ^5^ Includes secondary diagnostic outcome. ^6^ Neonatal death prior to diagnostic testing, unable to locate infant, or parent or guardian refusal of diagnostic testing.

**Table 4:** 2018–2019 Diagnostic Outcome Summary for 52,099 infants screened. ^1^

Classification	Abnormal Screen Results	Abnormal Diagnostic Outcomes
Metabolic Disorders	44	BIOT (4), CIT (1), GA1 (1), HMG deficiency (1), LCHAD/TFP deficiency (1), MCAD deficiency (5), MSUD (1), PKU (1)
Endocrine Disorders	42	CAH (2), CH (28)
Other Disorders	102	CF (10)
Total	188	55

^1^ Includes infants born out-of-province and registered in Alberta.
